# CHA_2_DS_2_-VA score for predicting ventricular arrhythmias and mortality in ICD patients with ischemic heart disease and dilated cardiomyopathy: results from a prospective cohort

**DOI:** 10.3389/fcvm.2026.1743535

**Published:** 2026-05-13

**Authors:** Benjamin Vögeli, Patrick Badertscher, Nicolas Schaerli, Christian Sticherling, Michael Kühne, Felix Mahfoud, Philipp Krisai, Beat Schär

**Affiliations:** Department of Cardiology, University Hospital Basel, University Basel, Basel, Switzerland

**Keywords:** dilated cardiomyopathy, implantable cardioverter-defibrillator, ischemic cardiomyopathy, risk prediction, ventricular arrhythmia

## Abstract

**Background and aims:**

The revised CHA_2_DS_2_-VA score facilitates stroke risk assessment in patients with atrial fibrillation. This study investigates the value of the CHA_2_DS_2_-VA for prediction of all-cause and cardiovascular mortality and ventricular arrhythmia (VA) in patients with ischemic heart disease (IHD) and dilated cardiomyopathy (DCM) undergoing implantable cardioverter-defibrillator (ICD) therapy.

**Methods:**

We analyzed data from 1,626 ICD recipients with IHD (*n* = 1,225) or DCM (*n* = 401) enrolled in a prospective registry at University Hospital Basel. Patients were stratified into low and high CHA_2_DS_2_-VA groups based on median score within each condition. Mortality and VA outcomes were assessed using Kaplan–Meier survival analysis and Cox proportional hazards models.

**Results:**

The median CHA_2_DS_2_-VA was 3 overall, 2 in DCM, and 4 in IHD patients. During a median follow-up of 7.5 years, 46% died, with an all-cause mortality rate of 6.1 per 100 patient-years. Higher CHA_2_DS_2_-VA scores were associated with increased all-cause and cardiovascular mortality in both IHD (HR 2.36, 95% CI 1.99–2.80, *p* < 0.001 for all-cause death) and DCM (HR 1.98, 95% CI 1.44–2.73, *p* < 0.001 for all-cause death). Each one-point increase in CHA_2_DS_2_-VA increased mortality risk (HR 1.4, 95% CI 1.34–1.47, *p* < 0.001 for all-cause death). No association was observed between CHA_2_DS_2_-VA and VA occurrence in the overall cohort or subgroups.

**Conclusion:**

The CHA_2_DS_2_-VA is strongly associated with all-cause and cardiovascular mortality in ICD patients with IHD and DCM, but does not predict VA. Thus, it may aid in mortality risk stratification but should not be used as a primary tool for arrhythmic risk assessment.

## Graphical Abstract

**Figure:**
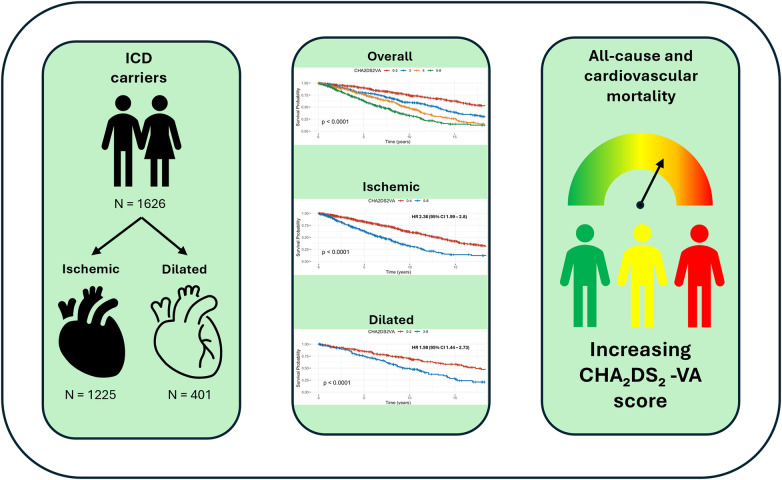

## 1 Introduction

The CHA_2_DS_2_-VA score is recommended for assessing stroke risk in patients with atrial fibrillation (AF) according to current guidelines (1, 2). However, emerging evidence suggests that the CHA_2_DS_2_-VA may have broader utility in predicting cardiovascular outcomes beyond AF, including mortality and ventricular arrhythmias (VA) in various patient populations (3–6).

Risk stratification remains challenging in patients with ischemic heart disease (IHD) undergoing implantable cardioverter-defibrillator (ICD) implantation (7). Identifying patients at higher risk for adverse outcomes such as mortality and VA is critical for optimizing clinical management.

Even more, in patients with dilated cardiomyopathy (DCM), the decision to implant an ICD for primary prevention remains complex and not well defined. While guidelines provide general recommendations, there is still uncertainty which DCM patients are at highest risk for life-threatening arrhythmias (8).

Given the association between the CHA_2_DS_2_-VA and cardiovascular outcomes in other populations, we hypothesize that this score may also be a valuable tool for predicting VA in ICD carriers, potentially aiding in more precise patient selection for ICD therapy.

By validating the predictive value of the CHA_2_DS_2_-VA in ICD carriers with IHD and DCM, this study aims to refine risk stratification models, ultimately improving clinical decision-making and patient outcomes.

## 2 Methods

### 2.1 Study design and participants

Since March 1994, all patients undergoing ICD implantation including CRT-defibrillators were included in a prospective ICD registry at the University Hospital of Basel. Data of 1,626 patients with either IHD or DCM were used for this analysis, with file closure in August 2024. The study was conducted in accordance with the declaration of Helsinki and was approved by the local ethics committee (Ethikkommission Nordwestschweiz, BASEC ID 2019-02190).

### 2.2 Outcomes

Co-primary outcomes were mortality and VA within the cohort and each of the groups. We assessed all-cause, cardiovascular (arrhythmic and non-arrhythmic), and non-cardiovascular death. VA was divided into three categories to provide granular outcomes reflecting clinically relevant sudden cardiac death risk levels: ventricular tachycardia (VT), fast VT (FVT) and ventricular fibrillation (VF). A VT event was defined with ventricular frequency <240/min and need for device therapy, a FVT event was defined with ventricular frequency ≥240/min and need for device therapy, and VF was defined as true ventricular fibrillation, thus a strong surrogate for sudden death. All events were confirmed by intracardiac electrograms. Only the first ever VA event and appropriate therapies were considered to define an event. All outcomes were adjudicated by the last author.

### 2.3 Clinical assessment and device mode

Data on cardiovascular risk factors, comorbidities, medication, and CHA_2_DS_2_-VA were assessed at implant. Mortality and VA during follow-up were determined in all patients up to time of file closure. Devices were implanted according to current practice and device modes are listed according to the NGB-code.

### 2.4 Statistical analysis

Baseline characteristics were stratified by IHD and DCM. Continuous data are presented as mean ± SD and categorical data as numbers (percentages). Categorical data were compared across groups using Pearsons's Chi-squared test and continuous data were compared using *t*-test. As a normal distribution of CHA_2_DS_2_-VA scores across the cohort was present we therefore dichotomized the CHA_2_DS_2_-VA into low/intermediate or high/very high scores based on the median CHA_2_DS_2_-VA of each group to ensure balanced sample sizes for comparison. We calculated mortality rates as the number of events divided by the total patient-years of follow-up and expressed per 100 patient-years; 95% confidence intervals (CI) were estimated using a Poisson distribution. Survival was analyzed using the Kaplan–Meier method, and the differences between the CHA_2_DS_2_-VA were compared using the log-rank test. Participants were censored at time of death, last follow-up or database closure (31/09/2026). For the overall cohort patients were divided in quartiles and for IHD and DCM groups according to the previously defined dichotomized approach. Cox proportional hazards regression models were then fitted to estimate the hazard ratios (HR) and 95% CI for the association between CHA_2_DS_2_-VA group and outcomes, using both dichotomized groups (high/very high vs. low/intermediate score) and CHA2DS2-VA as continuous predictor. *P*-values <0.05 were considered as statistically significant. All analyses were performed with RStudio version 4.4.2 (9).

## 3 Results

### 3.1 Baseline characteristics

**Figure 1:**
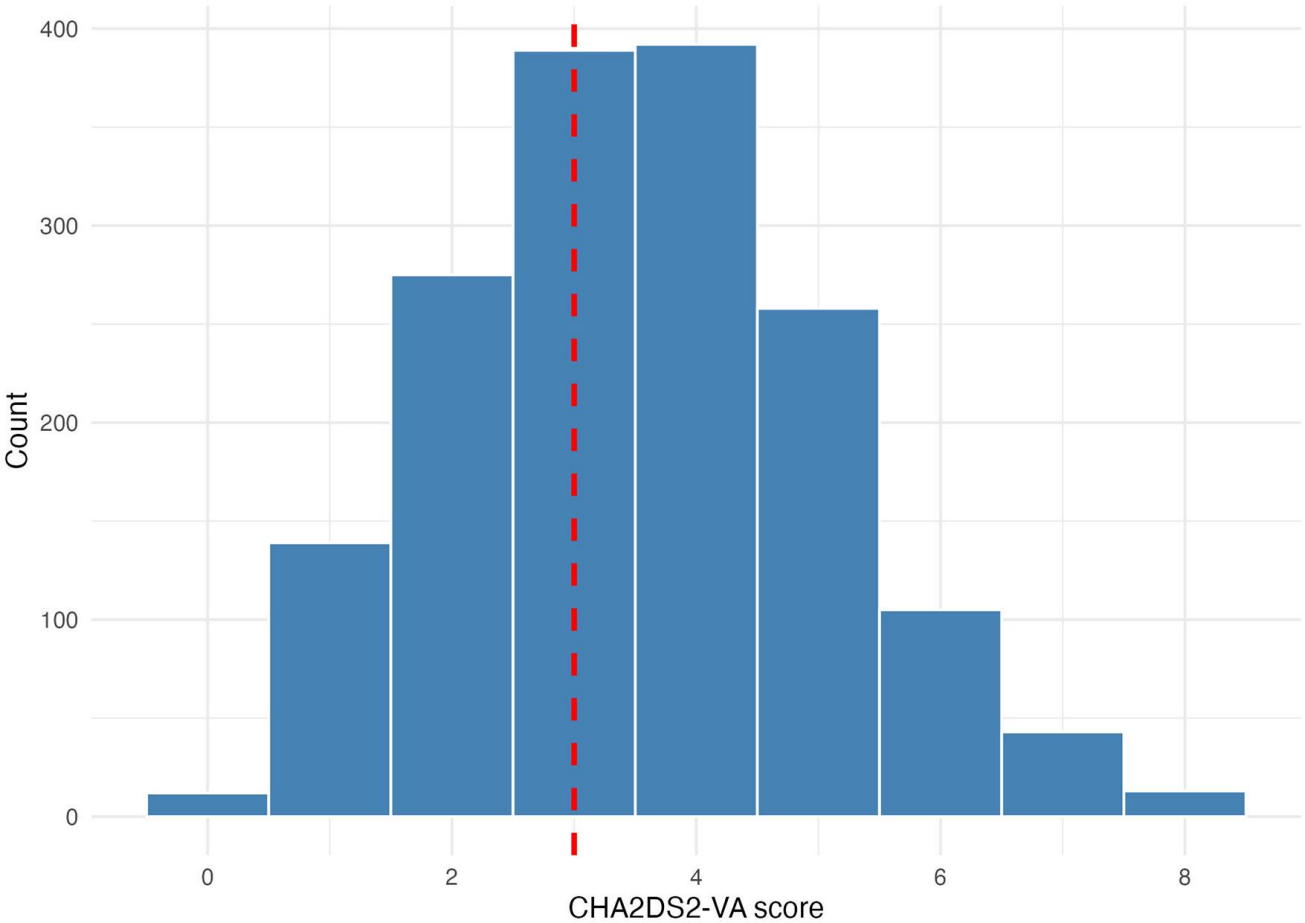
Histogram of CHA2DS2-VA score with red dashed lined indicating median CHA2DS2-VA score of the cohort.

Baseline characteristics are presented in Table 1. Age of the cohort was 64 (±13) years, 15% were female, and 71% had a history of acute myocardial infarction. Left ventricular ejection fraction (LV-EF) was 30% (±9) and 65% of all ICD were implanted for primary prevention. The median CHA_2_DS_2_-VA was 3 (Figure 1). Median follow up was 7.5 years. Patients with DCM were significantly younger (age 62 ± 13 years), there was a higher proportion of females (24%), LV-EF was lower and less cardiovascular risk factors were present, as compared to patients with IHD. The median CHA_2_DS_2_-VA was 2 in the DCM group and 4 in the IHD group. The most frequent device mode was VVI in patients with IHD (63%) and CRT in patients with DCM (50%).

**Table 1:** Baseline characteristics.

Baseline characteristics	*N* (%), mean (±SD)	*N* (%), mean (±SD)	*N* (%), mean (±SD)	*p*-value
	Overall (*n* = 1,626)	IHD (*n* = 1,225)	DCM (*n* = 401)	
Age (years)	64.0 ± 12.6	65.7 (±10.5)	61.6 (±13.2)	<0.001
BMI (kg/m^2^)	27.3 (± 4.7)	27.3 (±4.5)	27.1 (±5.0)	0.46
Male sex	1,387 (85.4%)	1,082 (88.3%)	304 (75.8%)	<0.001
Myocardial infarction	1,145 (70.5%)	1,142 (93.2%)	3 (0.7%)	<0.001
PCI	848 (69.2%)	843 (68.8%)	5 (1.2%)	<0.001
Atrial fibrillation at implant	195 (12%)	130 (10.6%)	65 (16.2%)	0.004
Stroke/TIA	178 (10.9%)	151 (12.3%)	27 (6.7%)	0.007
LVEF (%)	30 (± 9.4)	31.3 (±9.4)	26.2 (±8.1)	<0.001
Device mode				
VVI	932 (57.3%)	770 (62.9%)	162 (40.4%)	
DDD	205 (12.6%)	168 (13.7%)	37 (9.2%)	
CRT	441 (27.1%)	242 (19.8%)	199 (49.7%)	
VDD	42 (2.6%)	40 (3.2%)	2 (0.5%)	
S-ICD	6 (0.4%)	5 (0.4%)	1 (0.2%)	
Hypertension	1,034 (63.6%)	859 (70.1%)	175 (43.6%)	<0.001
Diabetes mellitus	439 (27%)	362 (29.5%)	76 (18.9%)	<0.001
eGFR (mL/min/1.73 m^2^)	69.8 (± 25.6)	69.3 (±25.6)	71.3 (±25.3)	0.19
Systolic BP (mmHg)	118.9 (±19.9)	118.7 (±20.1)	119.3 (±19.6)	0.32
CHA_2_DS_2_-VA (Median)	3	4	2	
Follow-up (years, median)	7.5 (±5.7)	7.5 (±5.7)	7.8 (±5.7)	0.15
Medication				
ACEI/ARB	1,546 (95.1%)	1,159 (94.6%)	387 (96.5%)	
Betablocker	1,436 (88.4%)	1,088 (88.8%)	348 (86.8%)	
Mineralocorticoid antagonist^a^	1,101 (62.1%)	488 (61.9%)	196 (62.8%)	
SGLT2 inhibitor^b^	148 (78.3%)	121 (80.1%)	27 (71%)	
Diuretics (all types)	1,043 (64.2%)	771 (62.9%)	271 (67.6%)	

ACEI, angiotensin converting enzyme inhibitor; ARB, angiotensin II receptor blocker; BMI, body mass index; BP, blood pressure; eGFR, estimated glomerular filtration rate; LVEF, left ventricular ejection fraction; PCI, percutaneous coronary intervention; TIA, transient ischemic attack.

a Data available in 1,101 patients, percentages are in relation to available data.

b Data available in 189 patients, percentages are in relation to available data.

### 3.2 Mortality events

**Figure 2:**
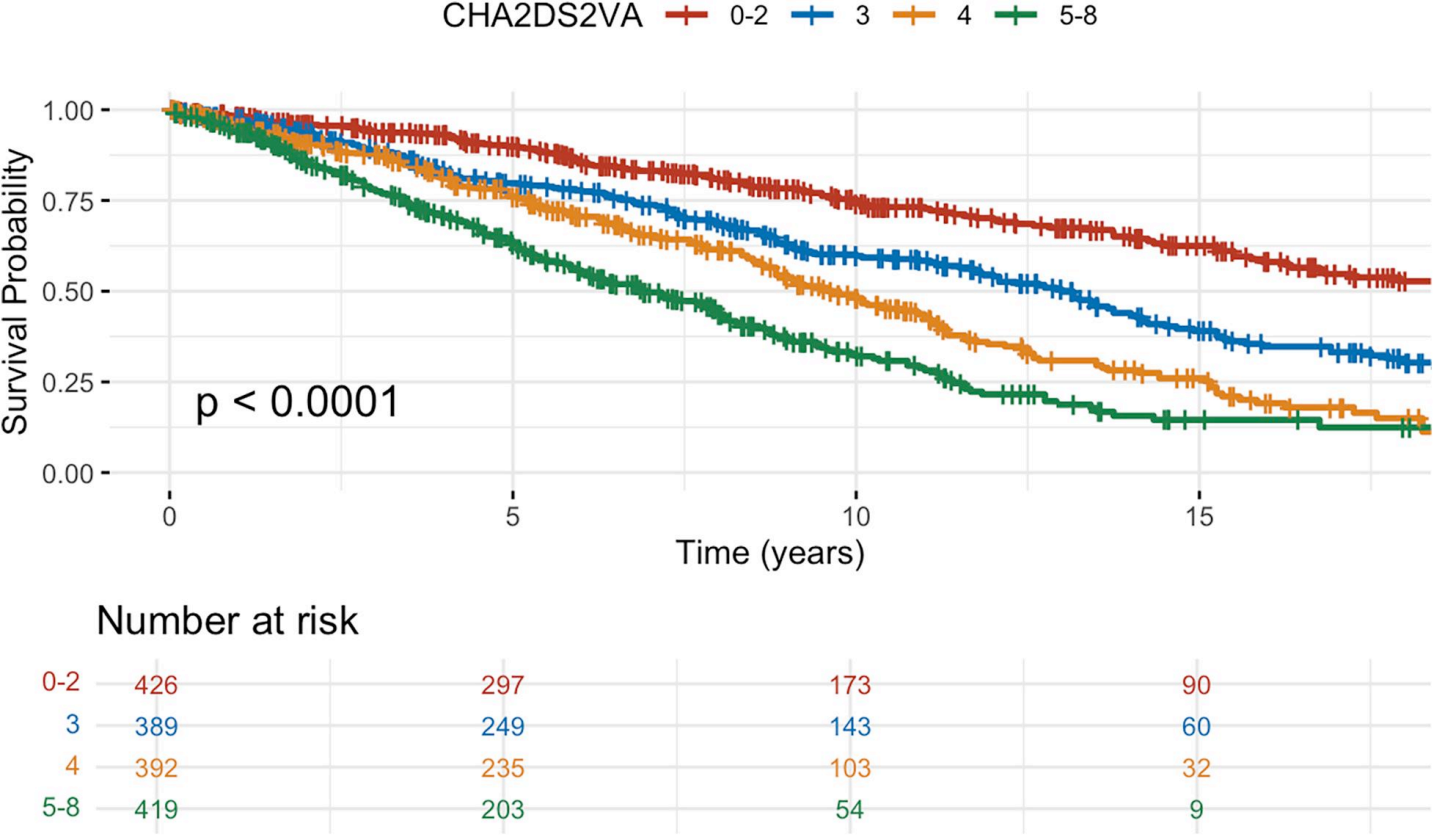
Kaplan–Meier survival curves of the study cohort for all-cause mortality stratified by CHA_2_DS_2_-VA score.

**Figure 3:**
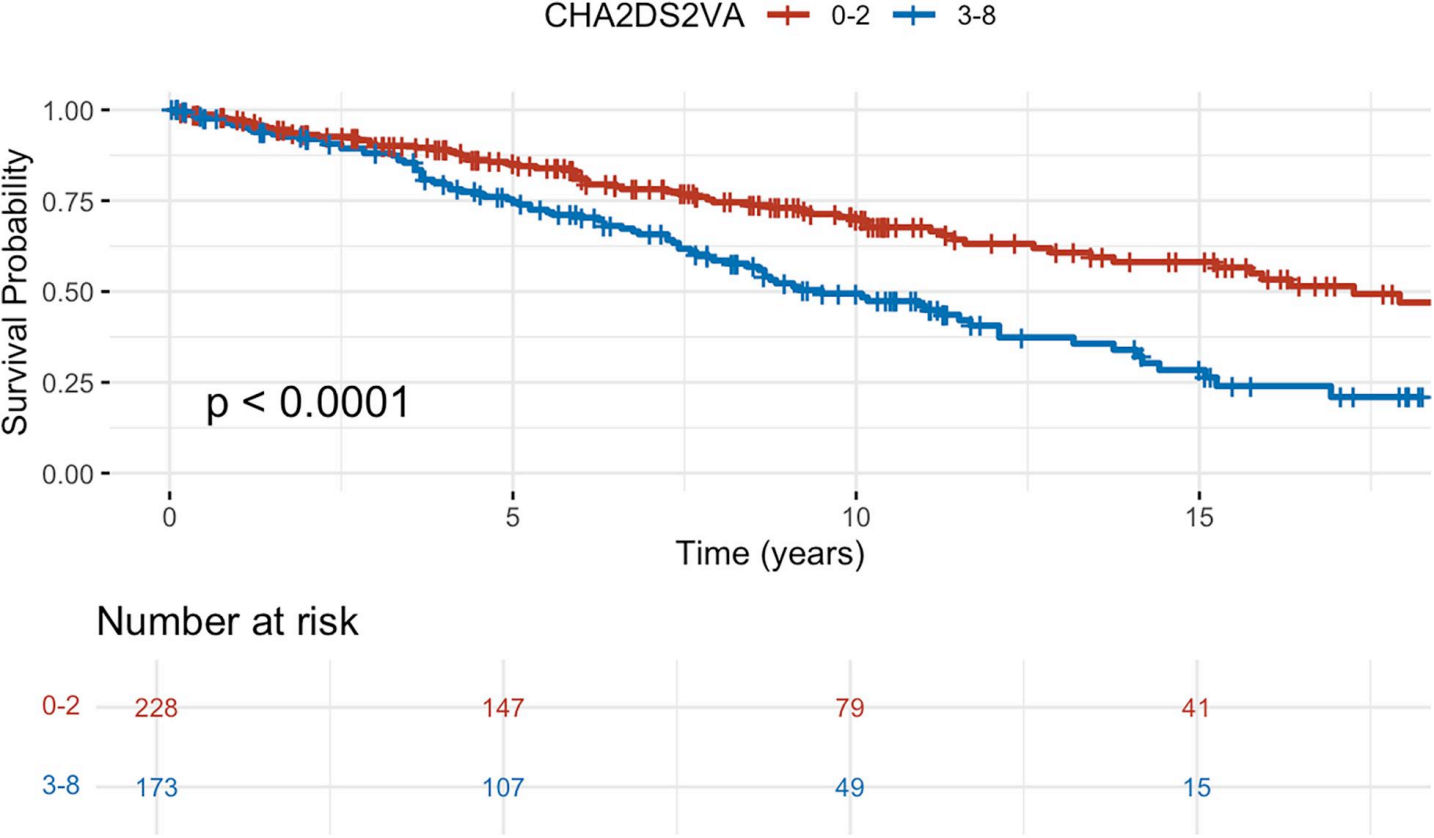
Kaplan–Meier survival curves for all-cause mortality of patients with dilated cardiomyopathy stratified by CHA_2_DS_2_-VA score split by the median to ensure reasonably balanced sample sizes.

**Figure 4:**
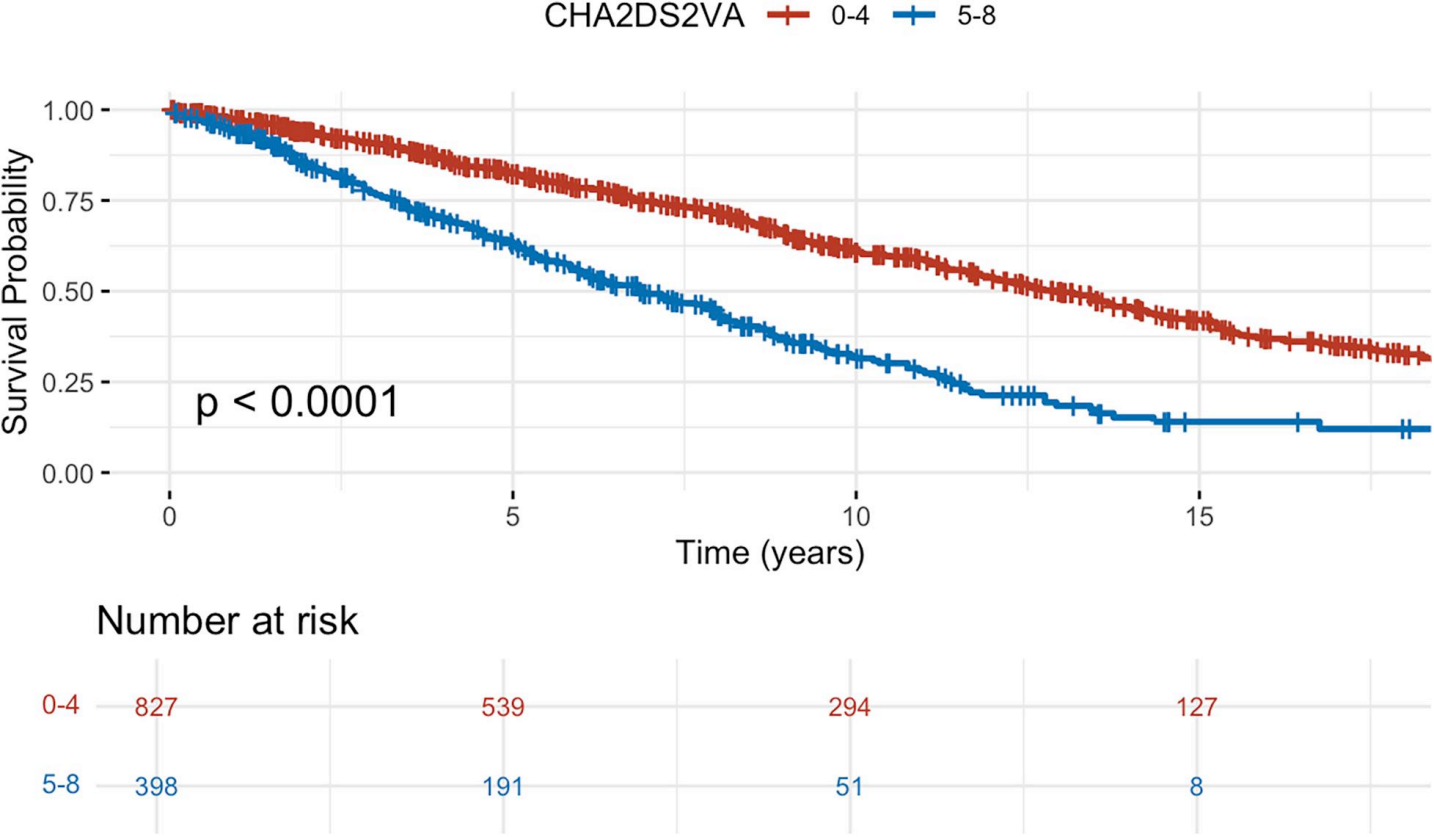
Kaplan–Meier survival curves for all-cause mortality of patients with ischemic heart disease stratified by CHA_2_DS_2_-VA score split by the median to ensure reasonably balanced sample sizes.

All-cause mortality was 46% and cardiovascular mortality was 22% over the median follow up time. The all-cause mortality rate was 6.1 per 100 patient years in the overall cohort, 6.5 among patients with IHD and 5.0 among those with DCM. All-cause mortality was significantly higher in patients with IHD, but no difference was seen for cardiovascular death. Among deaths from cardiovascular causes, the majority were non-arrhythmic. Survival probabilities showed significant differences when stratified by CHA_2_DS_2_-VA with highest survival probability in low CHA_2_DS_2_-VA (0–2) and lowest probability in high CHA_2_DS_2_-VA (5–8) (Figure 2, log-rank *p*-value <0.01). Similarly in patients with DCM, a higher CHA_2_DS_2_-VA (3–8) was associated with higher all-cause mortality compared to a lower CHA_2_DS_2_-VA (0–2) (Figure 3, HR 1.98, 95% CI 1.44–2.73, log rank *p* < 0.01). Mortality at 10 years was 30% in DCM patients with a lower CHA_2_DS_2_-VA, compared to 51% in patients with a higher CHA_2_DS_2_-VA. In patients with IHD, a higher CHA_2_DS_2_-VA (5–8) was associated with significantly increased mortality compared to a lower CHA_2_DS_2_-VA (0–4) (Figure 4, HR 2.36, 95% CI 1.99–2.8, log rank *p* < 0.01). 10-year mortality was 39% in IHD patients with a lower CHA_2_DS_2_-VA, compared to 69% in patients with a higher CHA_2_DS_2_-VA. When modelled as a continuous variable, each one-point increase in CHA_2_DS_2_-VA was associated with a higher risk of all-cause death in the overall cohort (HR, 1.40; 95% CI, 1.34–1.47; *p* < 0.001), in patients with ischemic heart disease (HR, 1.46; 95% CI, 1.38–1.55; *p* < 0.001), and in those with dilated cardiomyopathy (HR, 1.32; 95% CI, 1.18–1.48; *p* < 0.001). A similar association was observed for cardiovascular death (overall HR, 1.34; 95% CI, 1.25–1.43; *p* < 0.001; IHD HR, 1.40; 95% CI, 1.29–1.53; *p* < 0.001; DCM HR, 1.24; 95% CI, 1.06–1.46; *p* = 0.008). A summary of all events is presented in Table 2 and results from Cox proportional hazards regression models are presented in Tables 3, 4. The CHA_2_DS_2_-VA score had modest predictive ability for all-cause mortality (AUC 0.61) during long-term follow-up in this cohort.

**Table 2:** Summary of events.

Events	Overall	IHD	DCM	*p*-value
	*N* = 1,626	*N* = 1,225	*N* = 401	
All-cause death, *n* (%)^a^	746 (45.9%)	590 (48.2%)	156 (38.9%)	<0.001
All-cause death rate^b^ (95% CI)	6.1 (5.7–6.5)	6.5 (5.9–7.0)	5.0 (4.2–5.8)	
Cardiovascular death, *n* (%)	362 (22.3%)	282 (23.0%)	80 (20.0%)	0.34
Cardiovasculardeath rate^b^ (95% CI)	3.0 (2.7–3.3)	3.1 (2.7–3.5)	2.6 (2.0–3.2)	
Arrhythmic death, *n* (%)	90 (5.5%)	74 (6.0%)	16 (4.0%)	0.15
Non arrhythmic death, *n* (%)	272 (16.7%)	208 (17.0%)	64 (16.0%)	0.7
Non-cardiovascular death, *n* (%)	318 (19.6%)	251 (20.5%)	67 (16.7%)	0.46
Non-cardiovasculardeath rate^b^ (95% CI)	2.6 (2.3–2.9)	2.8 (2.4–3.1)	2.1 (1.7–2.7)	
Unknown death, *n* (%)	66 (4.0%)	57 (4.7%)	9 (2.2%)	0.68
VT, *n* (%)	501 (30.8%)	384 (31.3%)	117 (29.2%)	0.41
VT rate^b^ (95% CI)	4.1 (3.7–4.5)	4.2 (3.8–4.6)	3.7 (3.1–4.5)	
FVT, *n* (%)	329 (20.2%)	248 (20.2%)	81 (20.2%)	0.98
FVT rate^b^ (95% CI)	2.7 (2.4–3.0)	2.7 (2.4–3.1)	2.6 (2.1–3.2)	
VF, *n* (%)	128 (7.9%)	93 (7.6%)	35 (8.7%)	0.46
VF rate^b^ (95% CI)	1.0 (0.9 -1.2)	1.0 (0.8–1.3)	1.1 (0.8–1.6)	

FVT, fast ventricular tachycardia; VF, ventricular fibrillation; VT, ventricular tachycardia.

a Over median follow-up time.

b Per 100 patient years.

**Table 3:** Summary of Cox proportional analysis results with dichotomized groups.

Events	IHD^a^ (*n* = 1,225)		DCM^b^ (*n* = 401)	
	HR (95% CI)	*p*-value	HR (95% CI)	*p*-value
All-cause death	2.36 (1.99–2.8)	<0.001	1.98 (1.44–2.73)	<0.001
Cardiovascular death	2.2 (1.71–2.81)	<0.001	1.85 (1.18–2.89)	0.007
All VA	0.95 (0.79–1.14)	0.58	0.94 (0.68–1.29)	0.69
VT	1.0 (0.8–1.26)	0.98	1.31 (0.91–1.89)	0.15
FVT	0.84 (0.62–1.14)	0.26	0.68 (0.42–1.07)	0.09
VF	0.77 (0.47–1.25)	0.29	0.87 (0.44–1.74)	0.7

FVT, fast ventricular tachycardia; VA, ventricular arrhythmia; VF, ventricular fibrillation; VT, ventricular tachycardia.

a Patients were grouped in low (0–4) and high (5–8) CHA_2_DS_2_-VA.

b Patients were grouped in low (0–2) and high (3–8) CHA_2_DS_2_-VA and HR are shown are shown for high vs. low score.

**Table 4:** Summary of Cox proportional analysis results with CHA2DS2-VA score as continuous variable.

Events	Overall cohort (*n* = 1,626)		IHD (*n* = 1,225)		DCM (*n* = 401)	
	HR (95% CI)	*p*-value	HR (95% CI)	*p*-value	HR (95% CI)	*p*-value
All-cause death	1.4 (1.34–1.47)	<0.001	1.46 (1.38–1.55)	<0.001	1.32 (1.18–1.48)	<0.001
Cardiovascular death	1.34 (1.25–1.43)	<0.001	1.4 (1.29–1.53)	<0.001	1.24 (1.06–1.46)	0.008
All VA	1.01 (0.96–1.06)	0.67	1.01 (0.94–1.07)	0.86	0.98 (0.87–1.11)	0.79
VT	1.05 (0.96–1.11)	0.12	1.03 (0.95–1.1)	0.48	1.08 (0.95–1.24)	0.24
FVT	0.95 (0.88–1.03)	0.21	0.98 (0.89–1.07)	0.6	0.84 (0.7–1.0)	0.05
VF	0.96 (0.85–1.08)	0.46	0.95 (0.81–1.1)	0.48	0.98 (0.76–1.27)	0.9

DCM, dilated cardiomyopathy; IHD, ischemic heart disease; FVT, fast ventricular tachycardia; VA, ventricular arrhythmia; VF, ventricular fibrillation; VT, ventricular tachycardia.

### 3.3 Ventricular arrhythmia

A total of 958 first VA events (59%) were observed, ventricular tachycardia (VT and FVT) accounted for 87% of all events. The event rates were comparable between patients with IHD and DCM (Table 2). The incidence of VT was 4.2 per 100 patient years in the overall cohort, 4.1 among patients with IHD, and 3.7 among those with DCM. There was no association between the CHA_2_DS_2_-VA and occurrence of VA for the entire cohort, as well as for the two groups when analysed categorically (Table 3). Likewise, when analysed as a continuous variable, CHA_2_DS_2_-VA was not associated with risk of VA (overall HR 1.01, 95% CI 0.96–1.06, *p* = 0.67; IHD HR 1.01, 95% CI 0.94–1.07, *p* = 0.86; DCM HR 0.98, 95% CI 0.87–1.11, *p* = 0.79). Similar no associations were observed for subtypes of VA, including VT, FVT, and VF (Table 4). CHA_2_DS_2_-VA was not predictive for overall VA (AUC 0.54) or any type of VA (i.e., VT, FVT and VF, AUC 0.52, 0.58 and 0.53, respectively) in this cohort.

## 4 Discussion

In this large, unselected cohort of ICD patients with IHD and DCM we report two important main findings. First, the CHA_2_DS_2_-VA was associated with all-cause and cardiovascular mortality in this population. Second, the CHA_2_DS_2_-VA was not associated with any type of VA.

These findings extend prior research, which demonstrated an inverse relationship between the CHA_2_DS_2_-VASc and survival, by highlighting that a higher CHA_2_DS_2_-VASc is associated with increased mortality in ICD carriers, patients treated with cardiac resynchronization therapy devices, and in atrial fibrillation populations (4, 5, 10, 11). Earlier, the CHADS_2_ score showed good predictive accuracy for death and major cardiovascular events in syncope patients (12). This association may be explained by the fact that the CHA_2_DS_2_-VASc represents a composite of risk factors known to be associated with adverse outcomes and increased mortality (13–15). Interestingly, cardiovascular mortality rates were comparable between the groups, despite IHD patients being older and exhibiting more cardiovascular risk factors.

We did not find an association of the CHA_2_DS_2_-VA with occurrence of VA. In the MADIT-CRT population, Nof et al. described an inverse relationship between the CHA_2_DS_2_-VA and occurrence of VA and appropriate ICD shocks (5). However, in a population of AF patients with 42% of patients diagnosed with HF, Kuo et al. demonstrated an association of the CHA_2_DS_2_-VA with the risk of sudden cardiac death (SCD) and VA (3). These conflicting findings underline the complexity of identifying patients at risk for SCD and VA. While the CHA_2_DS_2_-VA did not predict VA in our cohort, other comorbidities such as AF may contribute to the risk of ventricular arrhythmias. AF has been shown to increase the risk of ventricular tachyarrhythmias 2–5-fold in both ischemic and non-ischemic cardiomyopathies (16, 17). Mechanistically, AF can promote ventricular arrhythmias through irregular ventricular activation, short-long-short sequences, and heterogeneous conduction, while also reflecting a shared arrhythmogenic substrate including myocardial fibrosis (18). Thus, AF may indicate both more advanced structural heart disease and a broader proarrhythmic substrate. Interestingly, in our cohort, the occurrence of VA did not differ between ischemic and non-ischemic patients, although this analysis was limited by considering only the first VT/VF event. Regardless, this finding aligns with previous reports showing similar arrhythmic risk for ischemic and non-ischemic cardiomyopathy (19). Nonetheless, the clinical benefit of ICD implantation is seemingly generally greater in ischemic compared to non-ischemic populations, with respect to mortality, need for advanced heart failure therapies and VA requiring ICD therapy (20).

In our study, the CHA_2_DS_2_-VA was associated with mortality not only when patients were stratified into low-/intermediate- and high-/very high-risk groups but also when analysed as a continuous variable. Each incremental point in the score conferred a substantially higher risk of both all-cause and cardiovascular death across the overall cohort and within the IHD and DCM subgroups. This gradient of risk suggests that the prognostic information conveyed by the CHA_2_DS_2_-VA is not limited to discrete thresholds but reflects a continuous relationship between comorbidity burden and survival. Our results align with previous attempts to stratify ICD benefit using clinical risk scores. Barsheshet et al. analyzed data from the MADIT-II trial and developed a simple five-factor model (age >70 years, NYHA class > II, AF, QRS ≥120 ms, and elevated blood urea nitrogen >26 mg/dL) to predict long-term survival benefit from prophylactic ICD implantation (21). They demonstrated that only patients in the low- and intermediate-risk categories showed a significant mortality reduction from ICD therapy, whereas those at highest baseline risk, largely due to comorbidities and non-arrhythmic deaths, did not benefit. This concept of competing risks, where high non-arrhythmic mortality attenuates ICD benefit, aligns with our observation that a higher CHA_2_DS_2_-VA identifies patients with increased overall and cardiovascular mortality but not with greater incidence of VA (22). Taken together, both studies underscore that mortality and arrhythmic risk are not necessarily congruent, and that clinical comorbidity burden may better reflect competing non-arrhythmic hazards rather than arrhythmic potential.

Even though a higher CHA_2_DS_2_-VA is associated with mortality, patient selection for primary prevention indication remains challenging. The primary goal is targeted ICD implantation in patients at highest risk for VA and SCD, while reducing unnecessary device implantation in those unlikely to benefit, particularly due to the competing risk of death before ICD therapy can be utilized (22–24). This complexity is further illustrated by prior analyses, such as the study by Goldenberg et al., which demonstrated a U-shaped relationship between baseline mortality risk and ICD benefit, with little or no survival advantage observed in patients at either very low or very high risk, and the greatest benefit confined to those at intermediate risk (25). In addition, substantial advances in guideline-directed medical therapy for heart failure over the past decade may further modify both arrhythmic risk and overall mortality, potentially limiting the performance and generalizability of traditional risk stratification models derived from earlier cohorts (26). This challenge is now being addressed in the context of post myocardial infarction patients in the PROTECT-ICD trial (NCT03588286) (27). By incorporating programmed ventricular stimulation and cardiac magnetic resonance imaging, the trial seeks to refine patient selection beyond LV-EF alone in the early phase after an ischemic event. In the context of non-ischemic cardiomyopathy, the DANISH trial did not show a mortality benefit of early ICD implantation compared to standard of care (28). The ReCONSIDER study (NCT 04246450) introduced a two-step, multifactorial approach for arrhythmic risk stratification in non-ischemic dilated cardiomyopathy. By combining non-invasive risk factor assessment with programmed ventricular stimulation, this strategy seeks to provide a comprehensive framework for identifying high-risk patients and thereby optimizing outcomes and resource utilization in this high-risk population (29). Our findings highlight the ongoing challenges in risk stratification and in accurately identifying patients who are most likely to derive benefit from ICD therapy.

Our findings have several implications for clinical practice. Given its association with all-cause and cardiovascular mortality, the CHA_2_DS_2_-VA aids in identifying high-risk patients who could benefit from closer follow-up, early initiation and rapid uptitration of guideline-directed medical therapy, or additional interventions, such as advanced monitoring strategies, including pulmonary artery pressure sensors, device-based heart failure diagnostics or structured home telemonitoring programs, to enable timely detection of clinical deterioration and more individualized management for improving long-term outcomes. However, given its limited predictive value for VA, the CHA_2_DS_2_-VA should not be used as a primary tool for assessing arrhythmic risk or guiding ICD implantation decisions.

The strengths of our study include a large, unselected cohort of ICD recipients with either IHD or DCM, along with a long median follow-up of 7.5 years. However, several limitations should be acknowledged. First, the observational nature of the study precludes causal inference. Second, the study was conducted at a single center and included predominantly male patients, which may limit generalizability to broader populations. Third, the burden of VA was not considered for this analysis. Since arrhythmic burden may offer additional prognostic information and better reflects the severity of underlying electrophysiologic instability, its exclusion could limit the comprehensiveness of our findings in characterizing the relationship between the CHA_2_DS_2_-VA and VA in this population.

## 5 Conclusion

The CHA_2_DS_2_-VA is associated with both all-cause and cardiovascular mortality in ICD recipients with IHD and DCM but does not predict arrhythmic complications. While useful for mortality risk stratification, the CHA_2_DS_2_-VA should not guide ICD implantation decisions. Further studies are needed to refine arrhythmic risk assessment in this population.

## Data Availability

The raw data supporting the conclusions of this article will be made available by the authors, without undue reservation.
